# Kawasaki disease: guidelines of Italian Society of Pediatrics, part II - treatment of resistant forms and cardiovascular complications, follow-up, lifestyle and prevention of cardiovascular risks

**DOI:** 10.1186/s13052-018-0529-2

**Published:** 2018-08-30

**Authors:** Alessandra Marchesi, Isabella Tarissi de Jacobis, Donato Rigante, Alessandro Rimini, Walter Malorni, Giovanni Corsello, Grazia Bossi, Sabrina Buonuomo, Fabio Cardinale, Elisabetta Cortis, Fabrizio De Benedetti, Andrea De Zorzi, Marzia Duse, Domenico Del Principe, Rosa Maria Dellepiane, Livio D’Isanto, Maya El Hachem, Susanna Esposito, Fernanda Falcini, Ugo Giordano, Maria Cristina Maggio, Savina Mannarino, Gianluigi Marseglia, Silvana Martino, Giulia Marucci, Rossella Massaro, Christian Pescosolido, Donatella Pietraforte, Maria Cristina Pietrogrande, Patrizia Salice, Aurelio Secinaro, Elisabetta Straface, Alberto Villani

**Affiliations:** 1 Bambino Gesù Children’s Hospital, Piazza S. Onofrio n. 4, 00165 Rome, Italy; 2 Università Cattolica Sacro Cuore, Fondazione Policlinico A. Gemelli IRCCS, Rome, Italy; 3 Giannina Gaslini Institute, Genoa, Italy; 4 Istituto Superiore di Sanità, Rome, Italy; 5 Università degli Studi di Palermo, Palermo, Italy; 6 Università degli Studi di Pavia, Pavia, Italy; 7 Policlinico Giovanni XXIII, Bari, Italy; 8 Ospedale S. Eugenio, Rome, Italy; 9 Università degli Studi Sapienza, Rome, Italy; 10 Università degli Studi Tor Vergata, Rome, Italy; 11 Ospedale Maggiore Policlinico, Milan, Italy; 12 Ospedale di Battipaglia, Salerno, Italy; 13 Università degli Studi di Perugia, Perugia, Italy; 14 University of Florence, Florence, Italy; 15 Università degli Studi di Torino, Turin, Italy; 16 Associazione Rari ma Speciali, Rome, Italy

**Keywords:** Kawasaki disease, Coronary artery abnormalities, Intravenous immunoglobulin, Aspirin, Personalized medicine, Innovative biotechnologies, Child

## Abstract

This second part of practical Guidelines related to Kawasaki disease (KD) has the goal of contributing to prompt diagnosis and most appropriate treatment of KD resistant forms and cardiovascular complications, including non-pharmacologic treatments, follow-up, lifestyle and prevention of cardiovascular risks in the long-term through a set of 17 recommendations.

Guidelines, however, should not be considered a norm that limits the treatment options of pediatricians and practitioners, as treatment modalities other than those recommended may be required as a result of peculiar medical circumstances, patient’s condition, and disease severity or individual complications.

## Background

In 2008 the first Italian Kawasaki disease (KD) Guidelines were published. During the subsequent years new data have been collected and updated treatment reports have been published.

Kawasaki: Linee Guida italiane.

### Scope

Scope of these revised Guidelines is to update evidence on the following topics:

- efficacy of therapy in the chronic phase of the illness and in its sequelae;
- short- and long-term follow-up;
- lifestyle and prevention of cardiovascular risks.

### Users

These Guidelines are directed to pediatricians who work in hospital, family pediatricians, and general practitioners who work with children affected by KD and for families of KD patients.

### Note for users

The clinical management of each KD patient requires the application of these recommendations based on the peculiar patient’s condition. We are pleased to publish updated diagnostic and therapeutic indications for both medical and paramedical staff as well as the most accurate information for families.

### Sponsorships

No person who participated in the drafting of these Guidelines has been sponsored.

### Dissemination

The text has been initially discussed during the Consensus Conference “Kawasaki Disease Italian Guidelines” in Rome during September 2015. The same text has been rediscussed in the 71st National Congress of the Italian Society of Pediatrics in Rome during June 2015, and finally approved in the 73th National Congress of the Italian Society of Pediatrics in Naples during June 2017.

### Updates

Future updates are planned within the next five years, or sooner, if the medical literature will reveal evidences showing that these Guidelines have become obsolete.

### Methods

Different experts in general pediatric medicine, cardiology, infectious diseases, rheumatology, immuno-allergology, dermatology, radiology, or biologists experts in cell oxidative stress have participated in writing these Guidelines. They have been supported by representatives of family associations. The team has been requested to systematically analyze the present literature about KD to define the following evidences about efficacy of therapies in the chronic phase and in its sequelae, and efficacy of short-term and long-term follow-up for KD patients.

The basic document was the previous Italian KD Guidelines, which were published in 2008 (*Marchesi A* et al. *Malattia di Kawasaki: Linee-Guida Italiane. Prospettive in Pediatria. 2008;38:266–83*). Additionally, further references from the last 8 years were considered, using PubMed and Cochrane databases.

The following key-words were used: “child”, “Kawasaki disease or Kawasaki syndrome”, “coronary arteries aneurysm and ectasia”, “echocardiography”, “multi-slide computed tomography”, “angiography”, “intravenous immunoglobulin”, “aspirin”, “corticosteroids”, “biological drugs”, “follow-up”, limiting the search to documents on humans and written in English or Italian. Heterogeneity of the available researches and their low number did not allow to perform a meta-analysis of each item. The recommendations of these Guidelines are based on the best evidences available. Stronger recommendations are based on high scientific quality data or, alternatively, on the consensus of experts. In the Evidence Based Medicine clinical Guidelines provide evidence levels based on the study design (Table 1) and reported effectiveness (Table 2).

**Table 1:** Level (class) based on study design, defined as follows

class I	meta-analyses or systematic reviews from randomized controlled trials
class II	single randomized controlled trials
class III	nonrandomized controlled trials
class IV	retrospective case-control studies
class V	number of cases without control group
class VI	opinions of committees of experts and authorities

**Table 2:** Classification (grade) based on effectiveness, defined as follows

grade A	highly recommended
grade B	recommended
grade C	recommended, but evidence is uncertain
grade D	non recommended
grade E	contraindicated

## Introduction

Nine years have passed since the first announcement of the Italian Guidelines for diagnosis and management of Kawasaki disease (KD) in a national journal, but recently many more data have become available in relationship with this acute systemic vasculitis occurring in childhood [1]. According to the 2012 “Revised International Chapel Hill Consensus Conference Nomenclature of Vasculitides” [2], KD involves small and medium-sized vessels in each organ and apparatus. In general terms, we can consider KD as a self-limited heterogeneous disease with unknown, probably multi-factorial, etiology, which primarily affects infants and children under 5 years of age [3, 4].

The most significant complications in KD are coronary artery aneurysms (CAA), but their overall incidence has been consistently reduced by prompt recognition of the disease and treatment with intravenous immunoglobulin (IVIG) within 10 days of fever onset [5–9]. Unfortunately, patients who show an insufficient response to IVIG are more prone to develop cardiac sequelae, such as dilation or stenosis of coronary arteries, myocardial infarction, and valvular lesions [10]. Diagnosis of KD is merely clinic, based on the diagnostic clinical criteria (shown in the Part I of these Guidelines). Actually, no clinical findings or tests can be considered specific for KD, and this circumstance makes diagnosis extremely challenging.

Goal of the second part of these Guidelines is to recommend the best practice tools in the management of complicated KD, based on the most actual scientific evidence, and improve the overall prognosis of the disease. These Guidelines have been created for pediatricians working in hospital, family pediatricians, and general practitioners or nurses managing children affected by KD and for families of KD patients.

Complications of KD can be distinguished in:

- Resistant forms of Kawasaki disease;
- Cardiovascular complications of Kawasaki disease;
- Other systemic complications of Kawasaki disease;
- Recurrent forms of Kawasaki disease.

## Resistant forms of Kawasaki disease

A resistant KD is defined by failure in the response to initial therapy with IVIG [11]. Patients may have a persistent or recrudescent fever (> 38 °C, axillary or rectal) after 36 h since the end of IVIG infusion. This event occurs in more than 10% of patients with KD [12, 13]. It is believed that IVIG non-responsiveness might reflect the severity of the underlying inflammation, explaining the increased incidence of CAA in this subset of patients. It would seem useful to stratify patients according to the risk to develop IVIG resistance and subsequent risk of developing CAA to undertake in those at higher risk a more aggressive initial treatment. Initial attempts were made by Asai and Kusakawa, followed by Iwasa, Harada and more recently by Kobayashi, Egami and Sano [14]. These predictive models have considered multiple factors, such as patient’s age, sex, duration of disease, white blood cell count, hematocrit, platelet count, C-reactive protein, transaminases, total bilirubin, albumin, sodium, but all models have shown a lower sensitivity in Western populations than in Japanese patients [15]. Unfortunately, there is currently no universally accepted scoring system to predict IVIG non-responsiveness, and for the Caucasian population there is no validated risk score yet.

## Cardiovascular complications of Kawasaki disease

The most relevant complications in KD are represented by CAA, and different remodeling phenomena will affect their outcome. The coronary artery dilation may start as ectasia, slight expansion (less than 5 mm in diameter), moderate dilation (from 5 to less than 8 mm) up to giant aneurysms (more than 8 mm in diameter).

As exposed in the Part I of these Guidelines, the concept of z-score was introduced several years ago to compare the coronary artery diameter to the body surface area and measure the standard deviation from the average in Z units (SD, z-score), using specific nomograms (www.parameterZ.com). This is recommended for the right coronary, left anterior descending, left main coronary arteries, and also for other vessels such as the aortic ring and ascending aorta. An accurate measurement of weight and particularly height is important to enable calculation of an accurate body surface area. According to the most updated data in the medical literature related to KD, we define the *absence of coronary artery involvement* if the z-score is < 2, the *presence of coronary artery dilation* if the z-score is between 2 and 2.5*,* and the *presence of coronary artery aneurysm* if the z-score is ≥2.5. This standard deviation system should be used in the initial diagnosis of CAA, when there is a suspicion of KD or when the selection may be more “coarse”, to avoid losing patients who may be at substantial risk in a near future (Table 3). Conversely, size criteria might be used in the follow-up of KD patients, particularly if localized coronary artery injuries have been found [16, 17].

**Table 3:** Classification of coronary artery abnormalities in the acute phase of Kawasaki disease and severity classification

No coronary artery involvement: z-score < 2	
Dilation of the coronary artery: z-score > 2 to < 2.5 SD	
Small aneurysm of the coronary artery: z-score ≥ 2.5 to < 5 SD	
Medium aneurysm of the coronary artery: z-score ≥ 5 to < 10 SD	
Giant aneurysm of the coronary artery: z-score ≥ 10 SD	

The majority of CAA occurs in the proximal segments and at the branch level. KD patients with normal coronary arteries or with mild ectasia at 6 weeks since disease onset have an overall good prognosis [6, 18, 19]. On the contrary, patients with persistent aneurysms are at risk of stenosis and/or thrombosis of the same arteries. Giant coronary aneurysms do not revert to a normal morphology. The repair of affected vessels occurs by wall remodeling without total “renstitutio ad integrum”, but with progressive intimal hyperplasia and fibrosis, that lead to stenotic changes of the coronary artery, with risk of thrombosis, myocardial ischemia, and sometimes even sudden death. Rarely new aneurysms appear later in patients with pre-existing aneurysms and, if this occurs, they represent post-stenotic dilations. In rare cases aneurysms can develop in the axillary or celiac arteries. Other different cardiovascular complications may develop less frequently in patients with acute KD, and include myocarditis, pericarditis or pericardial effusion with myopericarditis, valvular insufficiency, and, rarely, cardiac arrhythmias. A specific treatment may be required for these manifestations as well as for cardiac dysfunction or heart failure [20, 21].

Echocardiography remains the gold-standard to identify CAA during the acute phase of KD up to the first 6 weeks. However, computed tomography (CT) or magnetic resonance (MR) angiography can be required for an accurate risk stratification via evaluation of the vascular system, especially in growing children (see Part I, Chapter “Long-term” follow-up).

## Other systemic complications of Kawasaki disease

Other systemic KD complications are represented by anemia, hypoalbuminemia, electrolyte imbalance (especially hyponatremia), paralytic ileus, liver dysfunction, cholecystitis, seizures, diarrhea, vomiting, dehydration, and heart failure, even iatrogenic from IVIG infusion-related overload. Specific treatments are required for these complications. The exact cause of the severe hypotension in these patients is unknown, though probably due to several factors, i.e. inflammatory capillary leak, myocardial dysfunction and imbalance of cytokines. The occurrence of macrophage activation syndrome (MAS) has also been reported in KD, heralded by non-remitting fever, impaired liver function, hypofibrinogenemia, hypertriglyceridemia, hyperferritinemia, pancytopenia and frequently hemophagocytosis, that can be observed in bone marrow fine needle aspiration [22, 23]. Some authors have reported the presence of clinical symptoms and laboratory abnormalities compatible with MAS in 1.1% of KD patients if using the Ravelli’s diagnostic criteria and in 0.42% if using the 2009 hemophagocytic lymphohistiocytosis diagnostic criteria [24, 25]. Another complication is KD shock syndrome (KDSS), with similar symptoms to MAS, but with higher incidence, which was described by Kanegaye et al. in 2009 [26]: this disorder is associated with severely increased inflammatory markers, platelet consumption and increased risk of CAA, mitral regurgitation and prolonged myocardial dysfunction. In addition, patients with KDSS may be resistant to treatment with IVIG and may need additional anti-inflammatory treatments.

## Recurrent forms of Kawasaki disease

Recurrence of KD ranges from 1.4 to 3% (respectively from the Chinese and Japanese epidemiologic collection of studies available). Often KD symptoms are the same as for the first episode. A longlasting fever, IVIG resistance, elevated AST level, and reduced hemoglobin are all risk factors significantly associated with KD recurrence [27]. A recurring KD, sometimes incomplete and atypical, may be associated with higher incidence of CAA. Autoinflammatory syndromes should be considered for a comprehensive differential diagnosis in children with recurrence of KD [28, 29].

## Treatment of resistant forms of Kawasaki disease

Several second-line treatment options are available in the resistant KD, represented by additional IVIG infusions, intravenous methylprednisolone pulses, infliximab, ulinastatin, cyclosporine A, methotrexate, and plasmapheresis. Clinical trials for anakinra or canakinumab are ongoing. Randomized controlled trials that evaluated the effectiveness of different drugs apart from the second infusion of IVIG are few.

### Corticosteroids

Corticosteroids are usually administered in all vasculitides due to their immunosuppressive and anti-inflammatory effect, with the aim of blocking the potential risk of CAA in KD. Their use as first and second-line treatment is debated, because of the differences regarding the selection of patients (all patients versus high-risk patients) and the ethnicity (Caucasian versus Eastern populations) reported in different studies.

#### Methylprednisolone

##### Rational

The intravenous pulses of methylprednisolone have a quick immunosuppressive effect by blocking the inflammatory cytokines and give a lower risk of imbalance in electrolytes.

##### Indications

Intravenous pulses of methylprednisolone are indicated for patients with resistance to IVIG on the basis of symptoms and laboratory tests, and for patients resistant to IVIG after a first-line therapy. Intravenous pulses of methylprednisolone are off-label in KD.

##### Dosage

A single intravenous pulse of methylprednisolone at the dose of 30 mg/kg of body weight can be provided in combination with IVIG as a first-line treatment of high-risk KD patients; pulses can be provided for 3 days in the resistant cases.

Low-risk KD patients resistant to two previous infusions of IVIG can receive pulses of methylprednisolone (30 mg/kg/day) for 3 days.

##### Effectiveness

There is no evidence that a first-line treatment with IVIG combined with an intravenous pulse of methylprednisolone prevent CAA in all patients with KD, but only in those suspected to be resistant to IVIG. For IVIG-resistant KD patients the second-line treatment with intravenous methylprednisolone reduces the duration of fever, but the overall incidence of CAA remains similar [30–34].

##### Side effects

Intravenous methylprednisolone side effects are bradycardia, hypertension, hyperglycemia, and hypothermia. A close monitoring of vital signs, ECG, and blood pressure is required during pulse [35].

#### Prednisone

##### Rational

The main purpose of therapy with prednisone is to exert its anti-inflammatory effect, solving the vasculitis and blocking the potential risk of CAA: it has a more powerful action in comparison with cortisol, inhibits transcription of different inflammatory cytokine genes, and promotes transcription of anti-inflammatory cytokines and proteins.

##### Indications

Suspected IVIG-resistant patients on the basis of symptoms and laboratory tests or patients resistant to IVIG given as first-line therapy.

##### Dosage

If used in combination with IVIG in the first-line treatment the dose of prednisone is 2 mg/kg/day given intravenously in three doses; after fever and improvement of inflammatory markers prednisone can be administered orally. When CRP is normalized, prednisone can be continued for 5 days, then is reduced to 1 mg/kg/day in 2 doses for further 5 days, then at 0.5 mg/kg/day for further 5 days.

##### Effectiveness

The efficacy should be acceptable for high-risk patients, but different studies have shown contradictory results [36–38].

(Initial treatment with IVIG and prednisone for suspected IVIG-resistant KD: level of evidence class I, grade B; second-line treatment with prednisone for IVIG-resistant KD: level of evidence class III, grade B).

##### Side effects

Shock, infection, Legg-Calvé-Perthes disease, gastrointestinal perforation or bleeding, diabetes, cataracts, glaucoma, chorioretinopathy, pancreatitis, congestive heart failure, liver failure, arrhythmia, adrenocortical insufficiency, osteoporosis, myopathy, thrombosis, increased intracranial pressure, and seizures. Its use is contraindicated in patients with infections, heart or kidney failure, and history of previous heart attack.

### Biological drugs

Biologic agents are highly effective drugs targeting the presumed key-steps in the immune system, namely tumor necrosis factor (TNF)-α and interleukin (IL)-1, that are the triggering molecules in the KD-related vasculitis. Because of the paucity of safety and efficacy data, many considerations are derived from the experience with anti-TNF or anti-IL-1 drugs on short series of patients.

#### Infliximab

##### Rational

The serum concentration of TNF-α is elevated in patients with KD and many studies have reported a correlation between KD severity and CAA [39]. Infliximab (IFX), a chimeric monoclonal antibody against TNF-α, suppresses inflammation by blocking the action of TNF-α only, reducing the levels of interleukin-6, CRP, and TNF-α soluble receptor, reducing the severity of vasculitis, as demonstrated in animal models [40–42]. IFX may cause the production of antibodies in patients undergoing repeated doses, and lead to allergic reactions or loss of efficacy.

##### Indications

KD patients resistant to IVIG. IFX use is off-label for KD.

##### Dosage

Single intravenous dose of 5 mg/kg of body weight in 200–500 ml of saline solution for at least 2 h.

IFX has a half-life of about 9.5 days, and it has not been established a lower age limit for use. No absolute safety studies are available for infants and newborns.

##### Effectiveness

Many studies confirm IFX short-term efficacy in both IVIG-resistant and corticosteroid-resistant KD patients, and also its safety, in about 80% of cases [43–52]. In particular, the incidence of CAA might be reduced if infliximab is used within the 10th day of disease.

(Level of evidence for patients resistant to IVIG: class II, grade C).

##### Side effects

There are few data available for pediatric patients. Infusion-associated reactions, such as anaphylaxis, skin rashes, pruritus, headache, bronchospasm, and angioedema can appear; symptoms of hypersensitivity may occur even after 3 days, and include myalgia, fatigue, arthralgia, face and hand edema, dysphagia, urticaria, and headache. Exacerbation of heart failure and infectious diseases might be disclosed. Any ongoing infectious disease, and especially tuberculosis is an absolute contraindication to the use of IFX. Therefore, a screening strategy with family history, interferon-gamma release assays, and also chest X-ray might be useful to rule out tuberculosis. IFX should also be avoided in carriers of hepatitis B or C virus patients because of a risk of activation and acute exacerbation of chronic hepatitis. In addition, IFX should be postponed in case of MMR vaccination in the previous 2 months and V vaccination in the past month. There are no data about the development of malignant tumors.

#### Anakinra

##### Rational

Anakinra is a recombinant IL-1 receptor antagonist blocking the natural biological activity of IL-1, by competitively inhibiting binding of IL-1 and its receptor, and down-regulating many IL-1-mediated inflammatory reactions [53]. Based on a hypothetical model of KD as an autoinflammatory disease, it was assumed that anakinra might exert an antinflammatory effect on systemic and coronary artery inflammation in these patients [54].

##### Indications

KD patients resistant to IVIG. Anakinra use is off-label for KD.

##### Dosage

The suggested daily dose is 4–8 mg/kg of body weight for subcutaneously injections given for an overall period of 15 days since KD onset.

##### Effectiveness

There are only anecdotal reports about using anakinra in children with KD.

(Levels of evidence for patients resistant to IVIG: class V, grade C).

##### Side effects

They might involve the gastrointestinal tract (nausea, diarrhea, abdominal pain, hypertransaminasemia), respiratory system (upper respiratory infections, sinusitis, influenza-like illness, rarely pneumonia), or skin (bruising, urticarial-like lesions, local infections). Pain, erythema, and inflammation might often appear in the injection sites, usually during the first 4 weeks of therapy, then reversible within 1 or 2 weeks. Rare are allergic reactions, including anaphylaxis. Frequent is the report of neutropenia, moderate eosinophilia, or moderate thrombocytopenia.

#### Canakinumab

##### Rational

Canakinumab is a high-affinity human monoclonal antibody targeted at IL-1β, with no cross-reactivity with other members of the IL-1 family, including IL-1α, which has been authorized for the treatment of systemic juvenile idiopathic arthritis and different hereditary autoinflammatory syndromes [55, 56].

##### Indications

KD patients resistant to IVIG. Canakinumab use is off-label for KD.

##### Dosage

Single subcutaneous injection of 4 mg/kg for a body weight less than or equal to 40 kg.

##### Effectiveness

Clinical trials are currently in phase I to test canakinumab as a potential treatment for different disorders, such as chronic obstructive pulmonary disease, gout, and coronary heart disease.

(Level of evidence for KD patients resistant to IVIG: Class V, grade C).

##### Side effects

They can be of minor relevance: rhinitis, nasopharyngitis, nausea, vomiting, diarrhea, dizziness, headache, or a mild redness with swelling, warmth, or itching at the injection site. Sometimes they can be severe: allergic reactions (rash, hives, difficulty in breathing or swallowing, asthma, oral allergy syndrome), hemoptysis, and infections.

Other drugs which have been used in the drug-resistant forms of KD are ulinastatin, cyclosporine, and methotrexate. Table 4 summarizes mechanism of action, indication, dosage, side effects, and the levels of evidence for these drugs. However, the use of ulinastatin, cyclosporine, and methotrexate is off-label in KD.

**Table 4:** Other drugs used in the drug-resistant Kawasaki disease

	Ulinastatin [93, 94]	Cyclosporine A [95–97]	Methotrexate [98]
Mechanism of action	Inhibitor of human trypsin	Inhibition of calcineurin and increased activity of T cells	Folic acid antagonist, suppression of lymphoproliferation
Indications	Patients resistant to IVIG (also as initial treatment combined with IVIG and ASA in high-risk patients)	Patients resistant to IVIG (there are no studies in patients < 4 months of life)	Patients resistant to IVIG
Dosage	Optimal dosing in not yet determined in children, though in many studies The dosage is 5000 U/kg for 3–6 times per day (maximum dose: 50000 U); it should be given in a second vein or temporarily suspending IVIG infusion (as it becomes turbid in contact with other drugs)	4 mg/kg/day in 2 doses per os; in case of persistence of fever the dosage can be increased to 5–8 mg/kg/day; administered until CRP normalization or for 10–14 days	10 mg/m^2^/week per os, administered until fever disappears
Side effects	Anaphylactic shock, liver dysfunction, leukopenia, rash, itching, diarrhea, pain at the injection site	Hypercalcemia, hypomagnesemia, hirsutism, hypertension	Gastrointestinal signs, alopecia, risk of myelosuppression, anaphylaxis, infections, liver dysfunction, acute kidney failure
Level of evidence	Class III, grade C (First-line treatment with IVIG + ASA: class II, grade B)	Class V, grade C	Class V, grade C

In 2008 Onouchi et al. reported a gene responsible for susceptibility to KD, inositol 1,4,5-triphosphate 3-kinase C (*ITPKC*), which suppresses the activity of T cells and leads to oversecretion of multiple cytokines, if dysfunctional, suggesting that it may make KD patients more prone to IVIG resistance and development of CAA [57].

#### Plasmapheresis

##### Rational

Plasmapheresis removes cytokines and chemokines activated in KD directly from the bloodstream.

##### Indication

KD patients resistant to IVIG.

##### Dosage

The circulating plasma is replaced with albumin at 5%; the volume to replace corresponds to approximately 1–1.5 times the volume of circulating plasma, calculated according to the formula: [weight (kg)/13 × (1-Ht/100) × 1000] (Ht, hematocrit [in %]). A larger vein (femoral, subclavian, or jugular) should be used for albumin administration with a double lumen catheter; simultaneously heparin is administered at an intravenous bolus of 15–30 U/kg, then at 15–30 U/kg/hour.

##### Effectiveness

There are no prospective randomized studies to evaluate the efficacy of plasmapheresis in KD, but few retrospective ones comparing plasmapheresis to IVIG [58].

(Level of evidence: class V, grade C).

##### Side effects

Hypotension, hypovolemic shock, urticaria, anaphylactic reactions, hypocalcemia, nausea, vomiting, or bleeding disorders.

As already explained in the Part I of these Guidelines, we define KD high-risk patients those who are younger than 12 months, show elevated CRP, elevated serum aminotransferase, hypoalbuminemia, severe anemia at disease onset and those who might display early development of CAA, signs of macrophage activation syndrome or shock.

Recommendation 1.

Treatment of non-responder low-risk KD patients is a second bolus of IVIG. Pulses of methylprednisolone (30 mg/kg/day) for 3 consecutive days are suggested in the case of no response.

(III - B)

Recommendation 2.

Treatment of non-responder high-risk KD patients is a second bolus of IVIG + 3 pulses of methylprednisolone (30 mg/kg/day for 3 consecutive days, followed by prednisolone: 2 mg/kg/day), following gradual reduction of prednisolone dose and its suspension after the resolution of symptoms and normalization of CRP.

(I - B)

Recommendation 3.

Biological drugs may be used in IVIG-refractory KD patients.

(II - C)

**Fig. 1:**
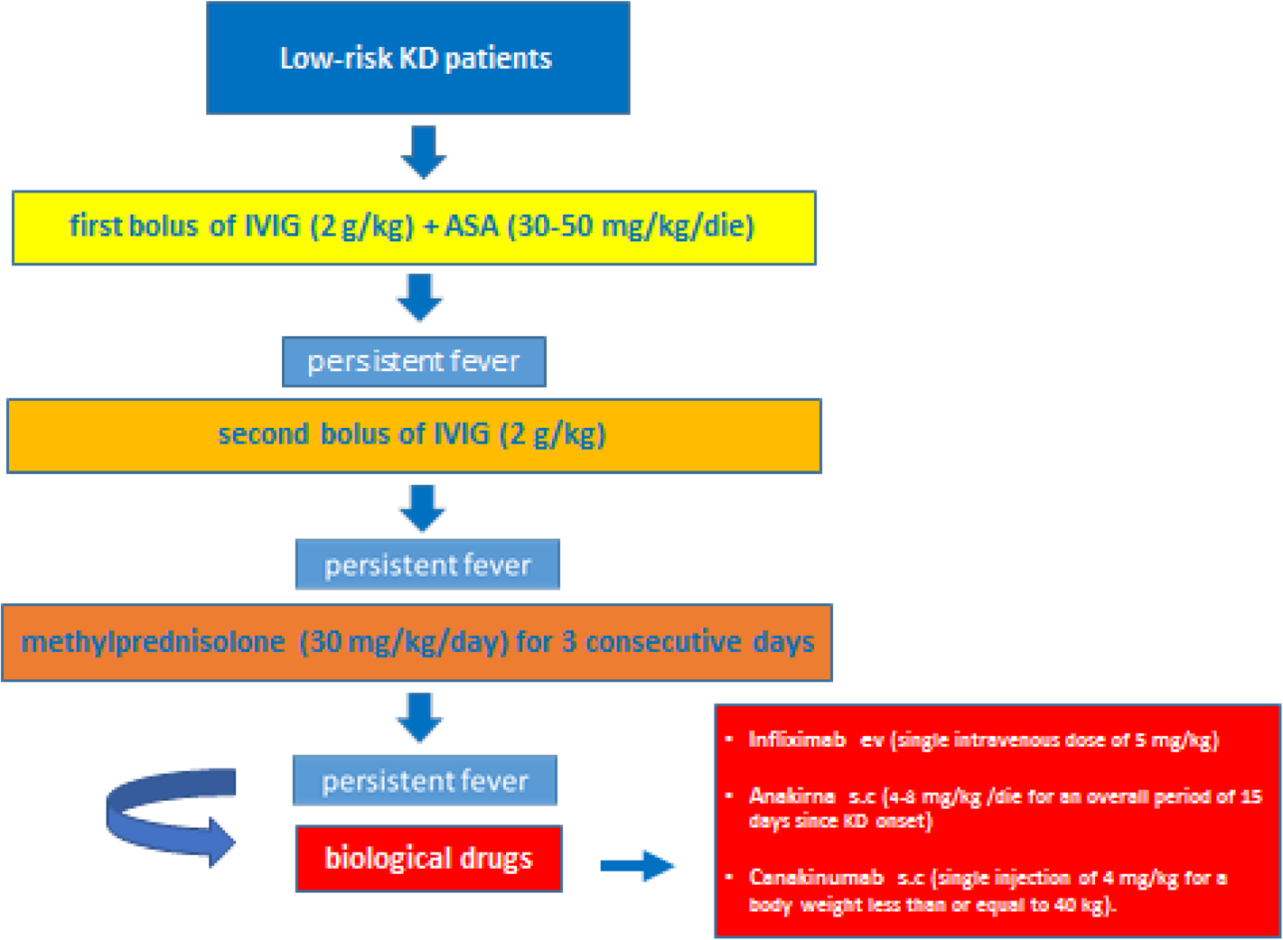
Treatment of low-risk KD patients

**Fig. 2:**
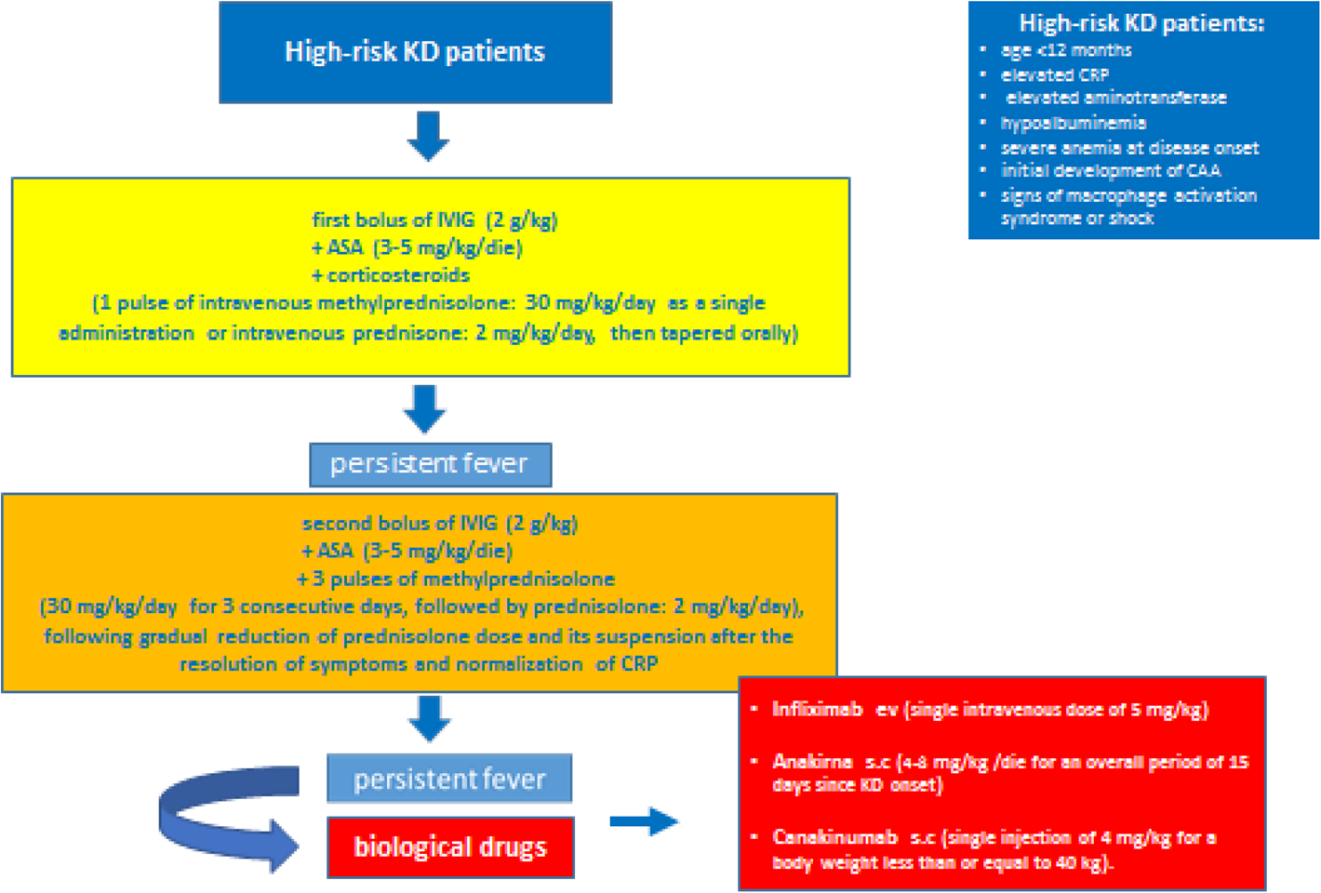
Treatment of high-risk KD patients

Finally, we have summarized the therapeutic recommendations for patients with KD, both low-risk and high-risk ones (Figs. 1 and 2).

## Treatment of the cardiovascular complications of Kawasaki disease

Regression of CAA depends on the initial size of the vascular dilation: about half of CAA resolves within a few years. In particular, a pseudo-normalization of luminal dimensions is observed within 1–2 years when aneurysms are small-sized and within 5 years in 80% of cases if medium-sized. Rarely giant aneurysms show a tendency to regress. Because of the repair process, however, a stenosis can occur in the incoming or outgoing aneurysmal area, secondary to intimal hyperplasia or thrombotic occlusion, with risk of myocardial ischemia, heart attack, and unexpected death [59]. Long-term therapy in KD patients with CAA aims at prevention of cardiovascular sequelae, reduction of both frequency and severity of heart attacks, and improvement of patient’s quality of life [60–62]. Such therapy depends on the severity and extension of coronary artery involvement. In the absence of “evidence-based” studies, recommendations have been drafted by pediatric retrospective studies, case reports, and extrapolation from experiences in adults.

Platelet activation is critical in all KD phases, therefore, in case of persistent CAA, a chronic use of antiplatelet low-dose ASA (3–5 mg/kg/day) must be provided, optionally associated with other antiplatelet and/or anticoagulant and/or anti-angina drugs, according to the size of aneurysms, flow characteristics, and presence or absence of myocardial ischemic changes.

### Other anti-platelet drugs

#### Clopidogrel

Mechanism of action of clopidogrel is based on the inhibition of platelet aggregation induced by ADP, and antiplatelet action is usually achieved with low doses of clopidogrel (0.2 mg/kg/day) in children aged < 24 months. Unfortunately there are no data related to children aged ≥25 months and the dose suggested is 1 mg/kg/day (the maximum adult dose is 75 mg/day). In case of ASA allergy or varicella (either wild disease or vaccination against varicella) clopidogrel might be an alternative to ASA [63]. Side effects of clopidogrel include malaise, myalgia, headache, dizziness, gastrointestinal symptoms, rash, itching, thrombotic thrombocytopenic purpura, and bleeding tendency. Clopidogrel is off-label in KD.

### Anticoagulants

In KD patients anticoagulants are indicated in case of medium and giant aneurysms and in cases complicated by thrombosis. Warfarin is the most widely used drug. Both in urgency and at the beginning of anticoagulant therapy warfarin should be associated with intravenous continuous infusion of heparin, administered at a full anticoagulant dose (monitoring the PTT, targeted between 60 and 85″ and 1,5–2,5 times the normal) for at least 48 h and in any case until reaching a steady INR between 2 and 2.5 to avoid the paradoxical thrombosis caused by the depletion of protein C and S, induced by early therapy with warfarin; then heparin should be suspended. It is recommended to pay attention to the bleeding tendency induced by these drugs. Although no large-scale studies exist, related to the use of heparin and low molecular weight heparin (LMWH) in KD, their use can be considered both in infants, for whom INR examination is not easy, and in case of reintroduction of warfarin after eventual suspension (due to eventual surgery). However, subcutaneous injections are required twice a day. Particularly, enoxaparin is a LMWH with demonstrated safety and efficacy, frequently used in the pediatric age [64]. Fondaparinux, a new LMWH with a longer half-life, might be used in selected cases (Table 5).

**Table 5:** Anticoagulant drugs used in Kawasaki disease

	Warfarin	Non-fractionated heparin	Low molecular weight heparin (LMWH)
Mechanism of action	Block of synthesis of vitamin K-dependent coagulation factors (II, VII, IX and X)	Bond with AT-III and inhibition of II, VII, IX, X, XI, XII coagulation factors	Bond with AT-III and inhibition of II, VII, IX, X, XI, XII coagulation factors
Therapeutic indications	Medium to giant aneurysms, history of heart attack, history of intra-aneurysm thrombosis	Aneurysms with high thrombotic risk, before starting therapy with warfarin	Same as non-fractioned heparin
Dosage	Initial dose of 0.05–0.12 mg/kg/day, progressively increased over 4–5 days to obtain an INR between 2.0 and 2.5	Initial intravenous dose: 50 U/kg in 10 min or more, followed by 20–25 U/kg/hour to maintain aPTT between 60 and 85″	
Side effects	Bleeding (epistaxis, gum bleeding, intracranial and intra-abdominal hemorrhage), embryopathies (dysostosis, dyschondroplasia, microcephaly)	Hemorrhage, thrombocytopenia, hepatic dysfunction, rash, diarrhea, hair loss, osteoporosis	Same as non-fractionated heparin, but less osteoporosis
Interactions	Reduced efficacy with chlorophyll contained in green and yellow vegetables (with high contents of vitamin K), vitamin K-enriched milk, phenobarbital, carbamazepine, rifampicin; increased efficacy if breastfeeding, use of erythromycin, fluconazole, corticosteroids, amiodarone	None	None

### New oral anticoagulants

The new oral anticoagulants (e.g. dabigatran, rivaroxaban, apixaban) show the advantage of having a predictable clinical effect without requiring routine laboratory check, though they are off-label in the pediatric age and do not currently have a clear indication in KD.

Recommendation 4.

Anti-platelet prophylaxis must be based on low-dose ASA (3–5 mg/kg/day) associated with clopidogrel (0.2 mg/kg/day in patients aged < 24 months or 1 mg/kg/day if age ≥ 25 months, max 75 mg/day) in a single dose for KD patients with medium-sized aneurysms (≥5 mm and ≤ 7 mm or if z-score ≥ 7 and < 10) or in those with multiple and complex aneurysms.

(VI - C)

Recommendation 5.

It is reasonable to treat KD patients with giant aneurysms (≥8 mm), with or without stenosis, with low-dose ASA associated with warfarin (keeping INR targeted at 2.0–3.0).

(II-III - B)

Recommendation 6.

In infants or older children in whom warfarin is difficult to adjust, it is reasonable to treat giant aneurysms with ASA and LMWH.

(VI - B)

Recommendation 7.

In KD patients with relevant and/or extraordinary risk of thrombosis, e.g. those with giant aneurysms or a recent coronary artery thrombosis, triple therapy with ASA, clopidogrel, and warfarin or LMWH should be considered.

(VI - C)

### Anti-angina drugs and coronary artery vasodilators

In KD patients younger than 2 years, symptoms of angina are rare. The main purpose of the anti-angina therapy is reducing heart rate and cardiac workload, reducing pre- and afterload, and increasing the coronary artery flow. Drugs of this group are beta-blockers, calcium channel blockers and nitro-vasodilators (see Table 6). Beta-blockers are the first-choice drugs for stable exercise-induced angina, and in KD they may be administered when a history of previous heart attack is present both to prevent the reinfarction, and reduce long-term mortality. The selective β-1 blockers are recommended to avoid side effects in other organs. Calcium channel blockers are the first-choice drugs for angina, but their intravenous administration is contraindicated in newborns and infants [17]. The use of all the above-mentioned drugs is off-label in KD.

**Table 6:** Long-term therapy for patients with coronary artery aneurysms related to Kawasaki disease and anginal symptoms

Patients without anginal symptoms:	
- *patients without demonstrated ischemia*: antiplatelet drugs - *patients with demonstrated ischemia*: antiplatelet drugs + calcium channel blockers. Evaluate revascularization, according to the pediatric cardiologist’s opinion	
Patients with anginal symptoms:	
In addition to antiplatelet drugs:	
- *patients with exercise-induced angina*: nitro-vasodilators and/or calcium channel blockers - *patients with angina at rest or during sleep*: calcium channel blockers - *patients with angina in the night*: calcium channel blockers + nitro-vasodilators	

### Statins

Hydroxymethylglutaryl coenzyme-A reductase inhibitors (statins) are a cornerstone of therapy for the primary and secondary prevention of atherosclerotic cardiovascular events in adults. They reduce low-density lipoprotein cholesterol and have potentially beneficial pleiotropic effects on inflammation, endothelial function, oxidative stress, platelet aggregation, coagulation, and fibrinolysis [1]. Although controversy continues concerning whether the vascular pathology of KD may lead to early atherosclerosis, statins could have a role in the long-term management of KD and an empirical low-dose treatment may be considered for patients with past or current aneurysms, regardless of age or sex [1].

## Treatment of coronary artery thrombosis

Patients with medium and giant aneurysms of coronary arteries and those in whom the aneurysm sizes rapidly increase over time are at greater risk of developing thrombosis and acute coronary syndrome. The majority of acute heart attacks caused by a thrombus in KD occurs within 2 years since disease onset. Coronary thrombosis may occur more frequently within the first 3 months of illness in these patients, with a peak incidence between 15 and 45 days after onset. It is therefore recommended to check frequently echocardiography during this period. A heart attack in childhood and in young children may be clinically silent or it may be associated with sneaky and nonspecific symptoms, such as unusual restlessness, vomiting, or shock. A sudden deterioration of cardiac function or a change in ECG findings should prompt the suspicion of coronary thrombosis.

Actually, more and more patients with KD-related coronary aneurysms reach adulthood, and this increases the risk of heart attacks caused by thrombosis of the aneurysm or progressive arterial stenosis caused by vascular remodeling. The goal of treatment in KD patients with coronary thrombosis is to restore the patency of the flow, “preserve” the myocardial tissue, and increase patient’s survival [65]. In the absence of randomized controlled trials recommendations about therapy of coronary thrombosis are derived from evidences in adult population-related studies, despite the etiology of stenosis is different, and from small series of pediatric cases.

Recommendation 8.

Coronary artery thrombosis with actual or imminent occlusion of the lumen should be treated with thrombolytic therapy or with interventional cardiac catheterization.

(I - C)

### Thrombolytic drugs

The most commonly administered thrombolytic therapy is intravenous tissue plasminogen activator (tPA, alteplase) [66–68].

#### Tissue plasminogen activator

##### Mechanism of action

Plasminogen activators are enzymes activating the conversion of plasminogen into plasmin, which can promote clot lysis.

##### Therapeutic indications

KD patients with acute heart attack or intra-aneurysmal thrombi, and KD patients with sudden extension of a thrombus in the coronary artery. Their use in KD patients is off-label.

##### Dosage

For alteplase: 29.0–43.5 × 10^4^ U/kg (administering 10% of the total dose over 1–2 min intravenously and infusing the remainder over 60 min).

##### Effectiveness

There are no data about the clinical efficacy and safety of thrombolytic agents in children, though we know that reperfusion following thrombolytic therapy is usually reached by approximately 70–80% of adult patients.

Recommendation 9.

Thrombolytic drugs have to be associated with low-dose ASA and low-dose heparin with careful monitoring for the bleeding risk.

(I - C)

Recommendation 10.

In children tPA (alteplase) is the first-choice thrombolytic drug, requiring to be associated with oral low-dose ASA and intravenous heparin (10 U/kg/hour).

(VI - A)

#### Other anti-platelet drugs

Less frequently, in the case of limited venous access, subcutaneously injected LMWH might be used. Considering the high risk of bleeding, unfractionated heparin should be a second-choice drug. Blood clotting test must be monitored daily, while fibrinogen level should be maintained > 100 mg/dL, and platelet count > 100.000/mm^3^ to minimize the bleeding risk during treatment with unfractionated heparin. In the case of significant thrombosis with high risk of occlusion, a combination of low-dose thrombolytic therapy may be also associated with a platelet aggregation inhibitor, i.e. abciximab (glycoprotein IIb/IIIa inhibitor) infused intravenously as a bolus of 0.25 mg/kg in 30 min, then followed by a continuous infusion of 0.125 mg/kg/minute (max 10 μg/min) for 12 h [69–71].

Recommendation 11.

Intravenous abciximab may be used as a bolus, followed by a continuous infusion for 12 h, in case of significant thrombosis with high risk of occlusion.

(VI - B)

Recommendation 12.

A small intracoronary mural thrombus “without immediate threat of occlusion”, detected by echocardiography in an early KD phase, may be treated with intravenous abciximab (10 U/kg/hour).

(VI - B)

## Non-pharmacological treatment of coronary artery thrombosis

### Invasive cardiologic procedures

Interventional catheterization procedures are limited by the large delivery systems for small patients, high risk of complications, and low efficacy, followed by high risk of reintervention. Coronary artery reperfusion both by invasive cardiologic interventional procedures and cardiac surgery can be considered after an initial unsuccessful pharmacological thrombolysis. Cardiologic procedures include percutaneous thrombolysis, percutaneous angioplasty, and rotational ablation [72–74]. CAA in KD are in general terms more difficult to treat than those in adults, and they often require that inflation pressure of the ball is greater, with higher risk of causing new aneurysms related to balloon dilation; the inflation pressure recommended is ≤10 atm. The application of a stent (“stenting”) is indicated in older children (≥13 years of age) in whom the calcification of CAA is relatively modest. The risk of development of new aneurysms after balloon dilation is lower in patients treated with the balloon dilation and stent compared to those treated with only balloon dilation, but the insufflation pressure of the ball should not be greater than 14 atm. A rotablator may be required to modify the shape of the lesion. Considering that surgery is performed in patients between 13 and 18 years of age, it is imperative that pediatric cardiologists share the clinical information with colleagues who will manage them into adulthood, for a more efficient transition. In order to determine the most appropriate interventional procedure it is desirable that physicians consider patient’s body size, coronary angiography findings, and the use of intravascular ultrasound (IVUS) [75].

#### Indications

- Patients with ischemic symptoms caused by significant coronary artery stenosis (≥75% of the luminal diameter).
- Patients in whom stenosis is significant (≥75% of the luminal diameter) in the absence of ischemic symptoms, but with instrumentally demonstrated ischemia (stress ECG, stress perfusion imaging, perfusion imaging stress).

#### Contraindications

- Patients with coronary ostial lesions.
- Patients with multi-vessel disease.
- Patients with significant coronary artery stenosis (≥75% of the luminal diameter) or occlusion of the contralateral coronary artery.

Recommendation 13.

First-choice cardiologic interventional treatment in patients with KD should be chosen on the experience of the center and its technical feasibility in the shortest possible time.

(V - C)

### Cardiac surgery

Percutaneous coronary angioplasty is associated with the risk of restenosis or occlusion in patients with KD, requiring often the use of coronary stents or alternative procedures, such as coronary artery bypass grafting (CABG) or rotational ablation. For children with persistent or progressive CAA who develop ischemic heart disease in early childhood a reliable option is represented by CABG using grafts from the internal mammary artery [76, 77].

#### Indications

- Patients with severe occlusive lesions of the left coronary artery.
- Patients with severe occlusive lesions in more [2 or 3] vessels.
- Patients with severe occlusive lesions in the proximal portion of the left anterior descending coronary artery.
- Patients with a compromised collateral circulation.
- Patients with previous history of a heart attack (to ensure secondary prevention of heart attacks).

In Japan the average age of children undergoing CABG is between 5 and 12 years, though it can be performed safely in younger children. The more frequent used surgical CABG technique is graft of the right or left thoracic mammary internal artery, which ensures good long-term patency (equal to 87% in general until 20 years). Other cardiac surgery associated with CABG may be required for the reduction of giant aneurysms in order to improve the coronary artery flow pattern and prevent the formation of thrombi caused by the increase of the “shear stress” on vessel walls [78, 79].

### Heart transplantation

A dozen of KD cases has been reported worldwide for heart transplantation, with an average age of 8.5 years, but also in subjects aged less than 4 months who experienced ventricular tachycardia or fibrillation [80].

#### Indications

- Patients with a significant left ventricular dysfunction.
- Patients with potentially lethal arrhythmias.
- Patients with significant lesions in the peripheral segments of the coronary arteries.

## Follow-up of patients with Kawasaki disease

### Short-term follow-up

Patients with KD must undergo a careful clinical monitoring of blood and instrumental exams in the long-term, but cardiological evaluations with ECG and echocardiography are crucial also in the short-term follow-up.

Recommendation 14.

Cardiologic evaluation with ECG and echocardiography should be performed at diagnosis of KD, to highlight eventual early CAA, then should be repeated at 2, 4 and 8 weeks after disease onset in patients who do not have developed aneurysms. In patients with CAA it is recommended to perform echocardiography 2 times a week during the period of rapid expansion of the dilation of coronary arteries, and at least once a week in patients with giant coronary aneurysms in the first 45 days after disease onset.

(VI - B)

Cardiological evaluation performed on the second month since disease onset allows to subdivide KD patients according to the cardiovascular impairment in different risk classes to establish a personalized follow-up. It is important an accurate measurement of coronary artery diameters by the evaluation of the relative z-scores.

### Long-term follow-up

Follow-up of KD patients must continue over time, especially for those who have presented CAA, considering that it is not possible to exclude remote complications even in the non-complicated cases. The aneurysmal lesions tend to regress (50–95%, according to case studies), mostly medium-sized and small-sized aneurysms. Such regression may be due to myointimal proliferation or thrombus organization and recanalization. Therefore, it is extremely important a careful long-term follow-up in KD patients with both persistent and regressed aneurysms with different timing and mode.

The class stratification risk, related to the risk of myocardial ischemia, established by the American Heart Association (AHA), is a useful tool for a standardized management of KD patients with regard to timing of controls, diagnostic tests, and therapeutic indications [6, 8].

It should be noted that risk classes of each patient with coronary artery impairment may vary over time because of morphological changes in the coronary arteries’ wall. The occurrence of thrombosis or stenosis associated with CAA, in fact, increases the risk of myocardial ischemia. Furthermore, the optimal follow-up of patients with regressed aneurysms remains still controversial since, even with the normalization of vessel diameter, morphological and functional alterations might persist. Indeed, the normal ultrasound coronary artery framework does not necessarily coincide with the normal endothelial function. This justifies continuing seriated controls, although less frequent, to trace the natural history of the disease, also regarding the possible risk of atherosclerosis. It is reasonable to use echocardiographic coronary artery luminal dimensions converted to body surface area-adjusted z scores to determine risk stratification of patients [81, 82]. Risk categories according to the AHA are shown in Table 7. Different therapies and follow-up modalities are recommended for each cardiovascular risk class.

**Table 7:** Cardiovascular risk classes in patients with Kawasaki disease

Class I	No abnormality of coronary arteries in the various phases of the disease
Class II	Transient coronary artery ectasia that disappears within 8 weeks
Class III	Single aneurysm of small-medium caliber between + 3 and + 7 SD in one or more arteries
Class IV	One or more aneurysms ≥7 SD, including multiple and complex giant aneurysms without any obstruction
Class V	Coronary artery obstruction at the angiography

Recommendation 15.

CLASS I

- ▪ Treatment with ASA for the first 8 weeks and in any case until a documented normalization of both platelet count and inflammatory markers.
- ▪ Cardiologic evaluations (ECG, echocardiogram, monitoring of blood pressure) and any blood chemistry tests with evaluation of lipid profile at 12 months since disease onset.
- ▪ No restriction of physical activity after the first 8 weeks.

CLASS II

- ▪ Treatment with ASA until normalization of both platelet count and inflammatory markers and disappearance of coronary artery lesions (also tortuosity/stiffness of the vessel walls), documented by two subsequent controls.
- ▪ Cardiologic evaluations (ECG, echocardiogram, monitoring of blood pressure) and blood chemistry tests with evaluation of lipid profile at 6 and 12 months after disease onset.
- ▪ Clinical reassessment of the eventual cardiovascular risk with physical activity counseling at 1 year since disease onset.
- ▪ No restriction of the physical activity after the first 8 weeks.

CLASS III

- ▪ Treatment with ASA until complete regression of the aneurysms documented by two subsequent negative controls.
- ▪ Cardiologic evaluations (ECG, echocardiogram, monitoring of blood pressure) and blood chemistry tests every 4–6 months, depending on the severity of lesions. If there is a complete regression of aneurysms documented by two subsequent negative controls, cardiologic examinations (ECG, echocardiogram, monitoring of blood pressure) annually in the first 3 years, then every 3–5 years (up to 18 years).
- ▪ Evaluation of myocardial perfusion every 2 years above the age of 10 (stress-ECG and/or stress ECO) with evaluation of the lipid profile.
- ▪ Coronary angiography or CT angiography if any myocardial ischemia is highlighted.
- ▪ After the first 8 weeks physical activity without restrictions (excluding athletic activities), guided by myocardial perfusion assessment tests in patients over 10 years.

CLASS IV

- ▪ Antiplatelet treatment (ASA + possible association with clopidogrel in selected patients) and any anticoagulant (warfarin in the giant aneurysms or LMWH in selected cases).
- ▪ Cardiologic evaluations (ECG, echocardiogram, monitoring of blood pressure) and blood chemistry tests every 4 months until a stable reduction of aneurysms is documented by two successive negative controls. Then, cardiology checks and blood tests annually with annual assessment of myocardial perfusion (stress ECG and/or stress echo or any stress MRI with contrast).
- ▪ Coronary angiography or coronary CT angiography in the first 6–12 months and thereafter when clinically indicated.
- ▪ Physical activity based on annual myocardial perfusion check; forbidden physical contact or collision sports/games.
- ▪ Counseling for pregnancy in female patients on anticoagulant treatment.

CLASS V

- ▪ Antiplatelet treatment (ASA + possible association with clopidogrel in selected patients) and anticoagulation with warfarin (or LMWH in selected cases).
- ▪ Cardiologic evaluations every 3 months with ECG and echocardiogram + possible Holter-ECG; annual assessment of myocardial perfusion (stress ECG and/or stress echo, stress MRI with contrast).
- ▪ Coronary angiography or coronary CT angiography to guide treatment options and, in case of myocardial ischemia, total body angio-CT.
- ▪ Coronary angiography or coronary CT angiography in the first 6–12 months, and thereafter when clinically indicated or suggested by non-invasive tests.
- ▪ Physical activity based on annual myocardial perfusion evaluation; forbidden physical contact or collision sports/games; avoid a sedentary lifestyle.
- ▪ Counseling for pregnancy in female patients on anticoagulant treatment.

(VI - C)

### Recommendations for a correct lifestyle and prevention of cardiovascular risks

Cardiovascular sequelae caused by KD differ substantially from the classic atherosclerosis from a pathological point of view. The endothelial dysfunction and chronic inflammatory reactions may occur even late in KD patients with giant or medium aneurysms, including those which have regressed, causing intimal hyperplasia and calcification if there is a localized stenosis. Although endothelial dysfunction is a precursor of atherosclerosis and autopsy studies seem to suggest that KD patients may have more severe atherosclerotic lesions, it is still unclear whether all KD patients have a higher risk for a clear progression to atherosclerosis [83–85]. It is reasonable to consider KD patients worthy of close monitoring for cardiovascular risks, evaluating blood pressure, body mass index, cholesterol, LDL, HDL, triglycerides, and promoting correct lifestyles (for instance, avoid smoking) and a regular physical activity with healthy eating [86, 87]. The use of statins is still debated and their role in KD is yet to clarify [88].

Recommendation 16.

Monitoring cardiovascular risk factors in KD patient requires body mass index evaluation, blood pressure control, and evaluation of the lipid profile (total cholesterol, LDL, HDL, triglycerides).

(VI - B)

#### Fitness certification for patients with a previous Kawasaki disease

##### Reference personnel for patients with a previous KD who want to perform *agonistic* sports (competitive sports or high-intensity activities) includes doctors specialized in sports medicine who work in the health systems or in authorized private structures; certification for *non-agonistic* sports can be provided by the family pediatrician and practitioner

In KD patients the evaluation for fitness certification is complicated by several reasons:

- there are non-subjective functional limitations (patients do not feel sick);
- cardiovascular risks depending on the coronary artery damage is difficult to quantify, and patients affected by giant aneurysms may show normal stress test when myocardial perfusion is evaluated;
- few data are available to define exactly risks caused by sport activity;
- it is difficult to correlate size of aneurysms to any certification for fitness, though giant or multiple aneurysms should be very carefully evaluated.

For the reasons above, specialized centres must certify physical fitness through multi-disciplinary evaluations. Activity restrictions depend on ongoing therapy (antiplatelet or anticoagulant drugs). Pediatricians can assist families in choosing which is the most appropriate sport.

Table 8 reports a classification of contact and collision sports, showing limited contact or non-contact sports, subdivided in high, mild, and less strenuous. Each patient on antiplatelet treatment must avoid contact and collision sports, while patients on anticoagulant therapy must also avoid limited contact sports [89].

**Table 8:** Sport classification according to the *American Academy of Pediatrics*

Contact/collision	Boxing, field hockey, ice hockey
	American football
	Motorcycle racing
	Martial arts, rodeo, soccer, wrestling
Limited contact	Baseball, basketball, bicycling, diving
	Field events (high jump, pole vault)
	Gymnastics, horse-back riding
	Ice roller skating
	Canoeing, fencing
	Running, swimming, tennis
	Race walking, weight lifting
Non-contact Highly strenuous	Skiing (cross-country, downhill, water)
	Softball
	Squash, team handball
	Volleyball
Non-contact Mildly strenuous	Badminton
	Curling
	Table tennis
Non-contact Non strenuous	Archery
	Golf
	Rifle range

Malattia di Kawasaki: Linee Guida italiane.

The following steps should be performed to certificate fitness: family and personal history, clinical examination and blood pressure control, resting 12-lead ECG, mono- and 2D-colour Doppler echocardiogram, tapis roulant stress test to determine stress tolerance, rhythm and heart frequency, and possible myocardial ischemia. In the presence of heart arrhythmias a dynamic 24 h-ECG must be performed. If all these examinations are normal, annual fitness certification can be produced for non-agonistic activities [90–92].

#### Indications for physical activity in patients with a previous Kawasaki disease

There are different indications for physical activity in children who have presented KD, which can be stratified according to various risk levels.

Recommendation 17.

Physical activity is recommended according to the following indications:

- risk level I e II: no restriction of physical activity (non-agonistic) if clinical assessment and instrumental tests are normal after 6–8 weeks.
- risk level III: no restriction of physical activity (non-agonistic) for children younger than 10–11 years after 6–8 weeks, then based on stress test each time a new certification is required. In selected cases the evaluation of myocardial perfusion might be indicated.
- risk level IV: physical activity is established on annual stress test and evaluation of myocardial perfusion. Contact/collision sports are not permitted because of the risk of bleeding. A coronarography must be performed if there is evidence of myocardial ischemia.
- risk level V: physical activity is established via six-monthly stress test with at least annual evaluation of the myocardial perfusion. Contact/collision sports are not permitted because of the risk of bleeding. Agonistic sports are not permitted, though a sedentary lifestyle should be avoided.

(VI - A)

## Discussion

Not applicable.

## Conclusion

This second part of practical Guidelines related to Kawasaki disease (KD) has the goal of contributing to the most appropriate treatment of resistant forms and KD-related cardiovascular/systemic complications, follow-up, lifestyle and prevention of long-term risks through a set of 17 recommendations.

These are based on the most actual scientific evidence, and their aim is improving the overall prognosis of the disease. We have refined the definition of resistant forms, recurrent forms, cardiovascular and systemic complications of KD. Concerning therapies, we have updated treatment of resistant forms (especially with corticosteroids and biological drugs), of cardiovascular complications (with anti-platelet and/or anticoagulants drugs)**,** and of coronary artery thrombosis (with both pharmacological and non-pharmacological approaches). Concerning follow-up of KD patients, we have analyzed short- and long-term suggestions for the follow-up, evaluating timing and type of different instrumental techniques, according to each cardiovascular risk class, introducing other advanced cardiovascular imaging techniques (i.e. computed tomography or magnetic resonance angiography). Although every clinical decision-making should be individualized to the specific patient with KD, herein we have pointed up recommendations for a correct lifestyle and overall prevention of cardiovascular risks, and finally we have reassessed indications for physical activity in patients with a previous KD.

## Abbreviations

ASA: Acetylsalicylic acid
AST: Aspartate aminotransferase
ATIII: Antitrombin III
CAA: Coronary artery aneurysms
CABG: Coronary artery bypass grafting
CRP: C-reactive protein
CT: Computed tomography
ECG: Electrocardiogram
IFX: Infliximab
IL: Interleukin
INR: International normalized ratio
IVIG: Intravenous immunoglobulin
KD: Kawasaki disease
KDSS: Kawasaki disease shock syndrome
LMWH: Low molecular weight heparin
MAS: Macrophage activation syndrome
MR: Magnetic resonance
PTT: Partial thromboplastin time
TNF: Tumor necrosis factor
tPA: Tissue plasminogen activator

## References

[1] McCrindle BW, Rowley AH, Newburger JW, Burns JC, Bolger AF, Gewitz M (2017). Diagnosis, treatment, and long-term Management of Kawasaki Disease, a scientific statement for health professionals from the American heart. Circulation..

[2] Jennette JC, Falk RJ, Bacon PA, Basu N, Cid MC, Ferrario F (2013). 2012 Revised International Chapel Hill Consensus Conference Nomenclature of Vasculitides. Arthritis Rheum..

[3] Esposito S, Rigante D, Principi N (2013). The role of infection in Kawasaki syndrome. J Infect.

[4] Rigante D, Tarantino G, Valentini P (2016). Non-infectious makers of Kawasaki syndrome: tangible or elusive triggers?. Immunol Res..

[5] Dajani AS, Taubert KA, Gerber MA, Shulman ST, Ferrieri P, Freed M (1993). Diagnosis and therapy of Kawasaki disease in children. Circulation..

[6] Newburger JW, Takahashi M, Gerber MA, Gewitz MH, Tani LY, Burns JC (2004). Diagnosis, treatment, and long-term management in Kawasaki disease: a statement for health professionals from the committee on rheumatic fever, endocarditis and Kawasaki disease, council on cardiovascular disease in the young. American Heart Association. Pediatrics..

[7] Falcini F, Capannini S, Rigante D (2011). Kawasaki syndrome: an intriguing disease with numerous unsolved dilemmas. Pediatr Rheumatol..

[8] De Rosa G, Pardeo M, Rigante D (2007). Current recommendations for the pharmacologic therapy in Kawasaki syndrome and management of its cardiovascular complications. Eur Rev Med Pharmacol Sci..

[9] Shulman ST, Rowley AH (2015). Kawasaki disease: insights into pathogenesis and approaches to treatment. Nat Rev Rheumatol..

[10] Rigante D, Valentini P, Rizzo D, Leo A, De Rosa G, Onesimo R (2010). Responsiveness to intravenous immunoglobulins and occurrence of coronary artery abnormalities in a single-center cohort of Italian patients with Kawasaki syndrome. Rheumatol Int.

[11] Yang HM, Du ZD FPP (2013). Clinical features of recurrent Kawasaki disease and its risk factors. Eur J Pediatr.

[12] Burns JC, Capparelli EV, Brown JA, Newburger JW, Glode MP (1998). Intravenous gamma-globulin treatment and retreatment of Kawasaki disease. US/Canadian Kawasaki Syndrome Study Group. Pediatr Infect Dis J..

[13] Wallace CA, French JW, Kahn SJ, Sherry DD (2000). Initial intravenous gamma-globulin treatment failure in Kawasaki disease. Pediatrics..

[14] Seki M, Kobayashi T, Kobayashi T, Morikawa A, Otani T, Takeuchi K (2011). External validation of a risk score to predict intravenous immunoglobulin resistance in patients with Kawasaki disease. Pediatr Infect Dis J..

[15] Rigante D, Andreozzi L, Fastiggi M, Bracci B, Natale MF, Esposito S (2016). Critical overview of the risk scoring systems to predict non-responsiveness to intravenous immunoglobulin in Kawasaki syndrome. Int J Mol Sci..

[16] McCrindle BW, Li JS, Minich LL, Colan SD, Atz AM, Takahashi M, et al. Pediatric Heart Network Investigators. Coronary artery involvement in children with Kawasaki disease: risk factors from analysis of serial normalized measurements. Circulation. 2007;116:174-9. 10.1161/CIRCULATIONAHA.107.690875 17576863

[17] Joint Working Groups: the Japanese Circulation Society, The Japanese Society of Kawasaki Disease, The Japanese Association for Thoracic Surgery, The Japan Pediatric Society, The Japanese Society of Pediatric Cardiology and Cardiac Surgery, The Japanese College of Cardiology. Guidelines for Diagnosis and Management of Cardiovascular Sequelae in Kawasaki disease (JCS 2013). Circ J. 2014;78:2521–62.

[18] Dillon MJ, Eleftheriou D, Brogan PA (2010). Medium-size-vessel vasculitis. Pediatr Nephrol..

[19] Brogan PA, Bose A, Burgner D, Shingadia D, Tulloh R, Michie C (2002). Kawasaki disease: an evidence based approach to diagnosis, treatment, and proposals for future research. Arch Dis Child..

[20] Wilson N, Heaton P, Calder L, Nicholson R, Stables S, Gavin R (2004). Kawasaki disease with severe cardiac sequelae: lessons from recent New Zealand experience. J Paediatr Child Health.

[21] Suzuki A, Miyagawa-Tomita S, Komatsu K, Nakazawa M, Fukaya T, Baba K (2004). Immunohistochemical study of apparently intact coronary artery in a child after Kawasaki disease. Pediatr Int..

[22] Stabile A, Bertoni B, Ansuini V, La Torraca I, Sallì A, Rigante D (2006). The clinical spectrum and treatment options of macrophage activation syndrome in the pediatric age. Eur Rev Med Pharmacol Sci..

[23] Wang W, Gong F, Zhu W, Fu S, Zhang Q (2015). Macrophage activation syndrome in Kawasaki disease: more common than we thought?. Semin Arthritis Rheum..

[24] Ravelli A, Magni-Manzoni S, Pistorio A, Besana C, Foti T, Ruperto N (2005). Preliminary diagnostic guidelines for macropphage activation syndrome complicating systemic juvenile arthritis. J Pediatr..

[25] Kang HR, Kwon YH, Yoo ES, Ryu KH, Kim JY, Kim HS (2013). Clinical characteristics of hemophagocytic lymphohistiocytosis following Kawasaki disease: differentiation from recurrent Kawasaki. Blood Res.

[26] Kanegaye JT, Wilder MS, Molkara D, Frazer JR, Pancheri J, Tremoulet AH (2009). Recognition of a Kawasaki disease shock syndrome. Pediatrics..

[27] Yang HM, Du ZD, Fu PP. Clinical features of recurrent Kawasaki disease and its risk factors. Eur J Pediatr. 2013;172:1641–7. 10.1007/s00431-013-2101-9 23887608

[28] Rigante D (2009). Autoinflammatory syndromes behind the scenes of recurrent fevers in children. Med Sci Monit..

[29] Rigante D (2017). A systematic approach to autoinflammatory syndromes: a spelling booklet for the beginner. Expert Rev Clin Immunol..

[30] Okada K, Hara J, Maki I, Miki K, Matsuzaki K, Matsuoka T (2009). Pulse methylprednisolone with gammaglobulin as an initial treatment for acute Kawasaki disease. Eur J Pediatr..

[31] Ogata S, Ogihara Y, Honda T, Kon S, Akiyama K, Ishii M (2012). Corticosteroid pulse combination therapy for refractory Kawasaki disease: a randomized trial. Pediatrics..

[32] Kobayashi T, Saji T, Otani T, Takeuchi K, Nakamura T, Arakawa H (2012). Efficacy of immunoglobulin plus prednisolone for prevention of coronary artery abnormalities in severe Kawasaki disease: a prospective, randomised, open, blinded-endpoint trial. Lancet..

[33] Newburger JW, Sleeper LA, McCrindle BW, Minich LL, Gersony W, Vetter VL (2007). Randomized trial of pulsed corticosteroid therapy for primary treatment of Kawasaki disease. N Engl J Med..

[34] Miura M, Tamame T, Naganuma T, Chinen S, Matsuoka M, Ohki H (2011). Steroid pulse therapy for Kawasaki disease unresponsive to additional immunoglobulin therapy. Paediatr Child Health..

[35] Miura M, Ohki H, Yoshiba S, Ueda H, Sugaya A, Satoh M (2005). Adverse effects of methylprednisolone pulse therapy in refractory Kawasaki disease. Arch Dis Child..

[36] Furukawa T, Kishiro M, Akimoto K, Nagata S, Shimizu T, Yamashiro Y (2008). Effects of steroid pulse therapy on immunoglobulin-resistant Kawasaki disease. Arch Dis Child..

[37] Etoom Y, Banihani R, Finkelstein Y (2013). Critical review of efficacy of immunoglobulin plus prednisone for prevention of coronary artery abnormalities in severe Kawasaki disease (RAISE study): a randomized, open-label, blinded-endpoints trial. J Popul Ther Clin Pharmacol..

[38] Millar K, Manlhiot C, Yeung RS, Somji Z, McCrindle BW (2012). Corticosteroid administration for patients with coronary artery aneurysms after Kawasaki disease may be associated with impaired regression. Int J Cardiol..

[39] Hii-Yuen JS, Duong TT, Yeung RSM (2006). TNF-α is necessary for induction of coronary artery inflammation and aneurysm formation in an animal model of Kawasaki disease. J Immunol..

[40] Saji T, Kemmotsu Y (2006). Infliximab for Kawasaki syndrome. J Pediatr..

[41] Hirono K, Kemmotsu Y, Wittkowski H, Foell D, Saito K, Ibuki K (2009). Infliximab reduces the cytokine-mediated inflammation but does not suppress cellular infiltration of the vessel wall in refractory Kawasaki disease. Pediatr Res..

[42] Nomura O, Fukuda S, Ota E, Ono H, Ishiguro A, Kobayashi T (2016). Monoclonal antibody therapy for Kawasaki disease: a protocol for systematic reviews and meta-analysis. Syst Rev..

[43] Burns JC, Mason WH, Hauger SB, Janai H, Bastian JF, Wohrley JD (2005). Infliximab treatment for refractory Kawasaki syndrome. J Pediatr..

[44] Stenbog EV, Windelborg B, Hørlyck A, Herlin T (2006). The effect of TNF-alpha blockade in complicated, refractory Kawasaki disease. Scand J Rheumatol..

[45] O’Connor MJ, Saulsbury FT (2007). Incomplete and atypical Kawasaki disease in a young infant: Severe, recalcitrant disease responsive to infliximab. Clin Pediatr..

[46] Oishi T, Fujieda M, Shiraishi T, Ono M, Inoue K, Takahashi A (2008). Infliximab treatment for refractory Kawasaki disease with coronary artery aneurysm. Circ J..

[47] Girish M, Subramaniam G (2008). Infliximab treatment in refractory Kawasaki syndrome. Indian J Pediatr..

[48] Burns JC, Best BM, Mejias A, Mahony L, Fixler DE, Jafri HS (2008). Infliximab treatment of intravenous immunoglobulin-resistant Kawasaki disease. J Pediatr..

[49] Brogan RJ, Eleftheriou D, Gnanapragasam J, Klein NJ, Brogan PA (2009). Infliximab for the treatment of intravenous immunoglobulin resistant Kawasaki disease complicated by coronary artery aneurysms: a case report. Pediatr Rheumatol..

[50] Singh S, Sharma D, Suri D, Gupta A, Rawat A, Rohit MK (2016). Infliximab is the new kid on the block in Kawasaki disease: a single-centre study over 8 years from North India. Clin Exp Rheumatol..

[51] Mori M, Imagawa T, Hara R, Kikuchi M, Hara T, Nozawa T (2012). Efficacy and limitation of infliximab treatment for children with Kawasaki disease intractable to intravenous immunoglobulin therapy: Report of an open-label case series. J Rheumatol..

[52] Shirley DA, Stephens I (2010). Primary treatment of incomplete Kawasaki disease with infliximab and methylpredonisolone in a patient with a contraindication to intravenous immune globulin. Pediatr Infect Dis J..

[53] Lopalco G, Cantarini L, Vitale A, Iannone F, Anelli MG, Andreozzi L (2015). Interleukin-1 as a common denominator from autoinflammatory to autoimmune disorders: premises, perils, and perspectives. Mediators Inflamm..

[54] Lee YH, Schulte DJ, Shimada K, Chen S, Crother TR, Chiba N (2012). IL-1β is crucial for induction of coronary artery inflammation in a mouse model of Kawasaki disease. Circulation.

[55] Yokota S, Kikuchi M, Nozawa T, Kanetaka T, Sato T, Yamazaki K (2015). Pathogenesis of systemic inflammatory diseases in childhood: Lessons from clinical trials of anti-cytokine monoclonal antibodies for Kawasaki disease, systemic onset juvenile idiopathic arthritis, and cryopyrin-associated periodic fever syndrome. Mod Rheumatol.

[56] Rigante D (2018). New mosaic tiles in childhood hereditary autoinflammatory disorders. Immunol Lett..

[57] Onouchi Y, Gunji T, Burns JC, Shimizu C, Newburger JW, Yashiro M (2008). *ITPKC* functional polymorphism associated with Kawasaki disease susceptibility and formation of coronary artery aneurysms. Nat Genet..

[58] Japan Apheresis Society Scientific Committee (2005). The present state of apheresis (results of the 2002 survey). Japan Apher Soc..

[59] Alpert JS, Thygesen K, Antman E, Bassand JP (2000). Myocardial infarction redefined: A consensus document of The Joint European Society of Cardiology/American College of Cardiology Committee for the redefinition of myocardial infarction. J Am Coll Cardiol..

[60] Yellen ES, Gauvreau K, Takahashi M, Burns JC, Shulman S, Baker AL (2010). Performance of 2004 American Heart Association recommendations for treatment of Kawasaki disease. Pediatrics..

[61] Satou GM, Giamelli J, Gewitz MH (2007). Kawasaki disease: diagnosis, management, and long-term implications. Cardiol Rev..

[62] Dominguez SR, Anderson MS (2013). Advances in the treatment of Kawasaki disease. Curr Opin Pediatr..

[63] Giglia TM, Massicotte MP, Tweddell JS, Barst RJ, Bauman M, Erickson CC (2013). Prevention and treatment of thrombosis in pediatric and congenital heart disease - A scientific statement from the American Heart Association. Circulation..

[64] Manlhiot C, Brandão LR, Somji Z, Chesney AL, MacDonald C, Gurofsky RC (2010). Long-term anticoagulation in Kawasaki disease: Initial use of low molecular weight heparin is a viable option for patients with severe coronary artery abnormalities. Pediatr Cardiol..

[65] Newburger JW, Takahashi M, Burns JC (2016). Kawasaki disease. J Am Coll Cardiol..

[66] Leaker M, Massicotte MP, Brooker LA, Andrew M (1996). Thrombolytic therapy in pediatric patients: a comprehensive review of the literature. Thromb Haemost..

[67] Albisetti M (2006). Thrombolytic therapy in children. Thromb Res..

[68] Williams MD (2010). Thrombolysis in children. Br J Haematol..

[69] Bachlava E, Loukopoulou S, Karanasios E, Chrousos G, Michos A (2016). Management of coronary artery aneurysms using abciximab in children with Kawasaki disease. Int J Cardiol..

[70] McCandless RT, Minich LL, Tani LY, Williams RV (2010). Does abciximab promote coronary artery remodeling in patients with Kawasaki disease?. Am J Cardiol..

[71] Williams RV, Wilke VM, Tani LY, Minich LL (2002). Does abciximab enhance regression of coronary aneurysms resulting from Kawasaki disease?. Pediatrics..

[72] Newburger JW, Fulton DR (2010). Coronary revascularization in patients with Kawasaki disease. J Pediatr..

[73] Gordon JB, Daniels LB, Kahn AM, Jimenez-Fernandez S, Vejar M, Numano F (2016). The spectrum of cardiovascular lesions requiring intervention in adults after Kawasaki disease. JACC Cardiovasc Interv..

[74] Suzuki A, Kamiya T, Ono Y, Takahashi N, Naito Y, Kou Y (1985). Indication of aortocoronary by-pass for coronary arterial obstruction due to Kawasaki disease. Heart Vessels..

[75] Kitamura S (2016). Pediatric coronary artery revascularization surgery: development and effects on survival, cardiac events and graft patency for children with Kawasaki disease coronary involvements. Iran J Pediatr..

[76] Akagi T (2011). Catheter interventions for Kawasaki disease: current concepts and future directions. Korean Circ J..

[77] Yuan SM (2012). Cardiac surgical procedures for the coronary sequelae of Kawasaki disease. Libyan J Med..

[78] Tsuda E, Miyazaki S, Yamada O, Takamuro M, Takekawa T, Echigo S (2006). Percutaneous transluminal coronary rotational atherectomy for localized stenosis caused by Kawasaki disease. Pediatr Cardiol..

[79] Peters TF, Parikh SR, Pinkerton CA (2002). Rotational ablation and stent placement for severe calcific coronary artery stenosis after Kawasaki disease. Catheter Cardiovasc Interv..

[80] Dionne A, Dahdah N (2018). Myocarditis and Kawasaki disease. Int J Rheum Dis.

[81] Muniz JC, Dummer K, Gauvreau K, Colan SD, Fulton DR, Newburger JW (2013). Coronary artery dimensions in febrile children without Kawasaki disease. Circ Cardiovasc Imaging..

[82] Mirhosseini SM, Asadollahi S, Fakhri M (2013). Orthotopic heart transplant for treatment-resistant cardiomyopathy in Kawasaki syndrome: report of a successful case. Prog Transplant..

[83] Dhillon R, Clarkson P, Donald AE, Powe AJ, Nash M, Novelli V (1996). Endothelial dysfunction late after Kawasaki disease. Circulation..

[84] McCrindle BW, McIntyre S, Kim C, Lin T, Adeli K (2007). Are patients after Kawasaki disease at increased risk for accelerated atherosclerosis?. J Pediatr..

[85] Cheung YF, Wong SJ, Ho MH (2007). Relationship between carotid intima-media thickness and arterial stiffness in children after Kawasaki disease. Arch Dis Child..

[86] Cohen JW (2013). Cardiovascular disease due to accelerated atherosclerosis in systemic vasculitides. Best Pract Res Clin Rheumatol..

[87] Cheung YF (2014). Vascular health late after Kawasaki disease: implications for accelerated atherosclerosis. Korean J Pediatr..

[88] Tremoulet AH (2015). The role of statins in inflammatory vasculitides. Autoimmunity..

[89] American Academy of Pediatrics Committee on Sports Medicine (1988). Recommendations for partecipation in competitive sports. Pediatrics..

[90] Graham TP, Driscoll DJ, Gersony WM, Newburger JW, Rocchini A, Towbin JA (2005). 36^th^ Bethesda Conference. Eligibility recommendations for competitive athletes with cardiovascular abnormalities. Task Force 2: Congenital Heart Disease. J Am Coll Cardiol..

[91] Thompson PD, Balady GJ, Chaitman BR, Clark LT, Levine BD, Myerburg RJ (2005). 36^th^ Bethesda Conference. Eligibility recommendations for competitive athletes with cardiovascular abnormalities. Task Force 6: Coronary Artery Disease. J Am Coll Cardiol..

[92] ESC Study Group of Sports Cardiology (2006). Recommendations for participation in leisure-time physical activity and competitive sports for patients with ischaemic heart disease. Eur J Cardiovasc Prev Rehabil..

[93] Kanai T, Ishiwata T, Kobayashi T, Sato H, Takizawa M, Kawamura Y (2011). Ulinastatin, a urinary trypsin inhibitor, for the initial treatment of patients with Kawasaki disease: As retrospective study. Circulation..

[94] Andreozzi L, Bracci B, D'Errico F, Rigante D (2017). A master role for neutrophils in Kawasaki syndrome. Immunol Lett..

[95] Suzuki H, Terai M, Hamada H, Honda T, Suenaga T, Takeuchi T (2011). Cyclosporin A treatment for Kawasaki disease refractory to initial and additional intravenous immunoglobulin. Pediatr Infect Dis J..

[96] Tremoulet AH, Pancoast P, Franco A, Bujold M, Shimizu C, Onouchi Y (2012). Calcineurin inhibitor treatment of IVIG-resistant Kawasaki disease. J Pediatr..

[97] Amazaki Y (2010). The calcineurin and NFAT system and its inhibition. Jpn J Clin Immunol..

[98] Lee TJ, Kim KH, Chun JK, Kim DS (2008). Low-dose methotrexate therapy for intravenous immunoglobulin-resistant Kawasaki disease. Yonsei Med J..

